# Experimental study on the quasi-static and dynamic tensile behaviour of thermally treated Barakar sandstone in Jharia coal mine fire region, India

**DOI:** 10.1038/s41598-024-54199-2

**Published:** 2024-03-04

**Authors:** Adarsh Tripathi, Mohammad Mohsin Khan, Anindya Pain, Nachiketa Rai, Mohd Ashraf Iqbal

**Affiliations:** 1 Department of Earth Sciences, Indian Institute of Technology, Roorkee, Uttarakhand 247667 India; 2 Department of Civil Engineering, Indian Institute of Technology, Roorkee, Uttarakhand 247667 India; 3 Geotechnical Engineering Group, CSIR-Central Building Research Institute, Roorkee, 247667 India

**Keywords:** Natural hazards, Solid Earth sciences

## Abstract

In the present study, the effect of mild to high-temperature regimes on the quasi-static and dynamic tensile behaviours of Barakar sandstone from the Jharia coal mine fire region has been experimentally investigated. The experimental work has been performed on Brazilian disk specimens of Barakar sandstone, which are thermally treated up to 800 °C. The quasi-static and dynamic split tensile strength tests were carried out on a servo-controlled universal testing machine and Split Hopkinson Pressure Bar (SHPB), respectively. Microscopic and mineralogical changes were studied through a petrographic investigation. The experimental results suggest the prevalence of both, static and dynamic loading scenarios after 400 °C. Up to 400 °C, the quasi-static and dynamic tensile strengths increased due to the evaporation of water, which suggests a strengthening effect. However, beyond 400 °C, both strengths decreased significantly as newly formed thermal microcracks became prevalent. The dynamic tensile strength exhibits strain rate sensitivity up to 400 °C, although it shows a marginal decline in this sensitivity beyond this temperature threshold. The Dynamic Increase Factor (DIF) remained constant up to 400 °C and slightly increased after 400 °C. Furthermore, the characteristic strain rate at which the dynamic strength becomes twice the quasi-static strength remains consistent until reaching 400 °C but steadily decreases beyond this temperature. This experimental study represents the first attempt to validate the Kimberley model specifically for thermally treated rocks. Interestingly, the presence of water did not have a significant impact on the failure modes up to 400 °C, as the samples exhibited a dominant tensile failure mode, breaking into two halves with fewer fragments. However, as temperature increased, the failure behaviours became more complex due to the combined influence of thermally induced microcracks and the applied impact load. Cracks initially formed at the centre and subsequently, multiple shear cracks emerged and propagated in the loading direction, resulting in a high degree of fragmentation. This study also demonstrates that shear failure is not solely dependent on the loading rate but can also be influenced by temperature, further affecting the failure mode of the sandstone.

## Introduction

The growing presence of subterranean structures at great depths has led to an escalating exposure of rock materials to elevated temperatures. Examples of such occurrences include the conversion of coal into gas in deep formations^1^, the deep geological processing of highly radioactive nuclear waste^2^, the exploration of geothermal energy^3^, and the study of land subsidence caused by underground coal mine fires^4^. The investigation into the mechanisms of damage in thermally treated rocks has been a captivating subject for researchers across various domains related to rock mass projects^5,6^.

In the context of dynamic loading, the mechanical properties of thermally treated rocks, such as compressive strength and tensile strength, are also influenced by the rate at which they are subjected to strain or load. During the last five years, several researchers have made efforts to comprehend how rocks behave dynamically under high-temperature conditions^7–10^. An et al.^7^ explored the dynamic testing of marble to ascertain both its uniaxial compressive strength and Brazilian tensile strength under varying temperatures. Their findings notably indicated that the strength of the rock augmented as the loading rate increased. An et al.^7^ reported progressive decrease in the strength of the rock with increasing temperature. Contrastingly, Meng et al.^8^ delved into investigating the strength of thermally treated sandstone, analyzing the impact of different impact speeds. Their study intriguingly suggested that varying impact speeds mitigated the adverse effects of temperature on sandstone strength. Furthermore, Ping et al.^9^ conducted dynamic compression tests on sandstone under real-time temperature conditions. Their conclusions highlighted the increasing brittleness of sandstone up to 400 °C, followed by a slight transition toward ductility up to 800 °C. In a related context, Zhao et al.^10^ studied the dynamic compressive strength of sandstone under high-temperature conditions. Their research revealed that the peak strength of sandstone exhibited an increase up to 100 °C. Although these diverse studies^7–10^ collectively contribute to understanding the complex behaviour of rocks under changing thermal and loading conditions, they also leave several open questions that need to be answered. Lindholm et al.^11^ established a correlation between the dynamic compressive strength, strain rate, and treated temperature of Dresser basalt. Zhang et al.^12^ experimented using SHPB to investigate the dynamic fracture of Fangshan marble and Fangshan gabbro at temperatures up to 330 °C, concluding that the dynamic fracture toughness is primarily determined by the loading rate. However, Yin et al.^13^ demonstrated that the dynamic fracture toughness of Laurentian granite decreases as the temperature rises to 850 °C. The weakening of strength can be attributed to the formation and opening of thermally induced microcracks^13–15^, as well as grain expansion, dehydration, phase transition, and recrystallization in rocks at elevated temperatures. Yao et al.^16^ found that decreasing dynamic tensile strength of Longyou sandstone with increasing temperatures, except at 450 °C due to the baking effect of clay minerals that may close the cracks and voids, which enhances the dynamic tensile strength. Fan et al.^17^ observed transitional temperatures before which parameters slightly decreased and after which they decreased drastically. Similarly, Xu et al.^18^ demonstrated 110 °C as the threshold temperature for the granite below which dynamic properties and energy absorption are improved as pre-existing microcracks close, providing a compact structure. However, above this threshold temperature, dynamic properties start to decrease with elevated temperature. Xu et al.^19^ investigated the thermal effect on rock fragmentation characteristics of Laurentian granite using a dynamic ball compression test and concluded that dynamic tensile strength is continuously decreasing with increasing temperature due to thermal damage. Furthermore, Zhang et al.^20^ found an increasing trend in the compressive strength of sandstone up to 400 °C, which then decreased with increasing temperature. Fan et al.^21^ evaluated the three cooling methods on the dynamic properties of sandstone and concluded that compressive strength increases up to 200 °C and sharply reduces afterwards for natural cooling due to the improved compactness of sandstone. However, compressive strength decreases linearly with temperature in rapid cooling and liquid nitrogen cooling. Furthermore, Yang et al.^22^ reported that the dynamic compressive strength of granite remains almost stable up to 450 °C and decreases thereafter. It is clearly evident from previous works^7–22^, that the mechanical properties of rocks under dynamic loading, especially when subjected to thermal treatment, exhibit intricate dependencies on several factors, including strain rate, temperature variations, and lithological composition. Earlier studies also highlighted how factors such as strain rate, temperature, and lithology impact dynamic properties such as compressive strength and fracture toughness. These findings indicate a complex interplay between temperature-induced effects, microcrack formation, and structural changes, showcasing both enhancements and deteriorations in mechanical behaviour across different rock types and temperatures, which needs to be further investigated through a well-planned and systematic experimental approach.

The evaluation of a rock's tensile properties holds significant importance when it comes to understanding and characterising the dynamic attributes of the rock. The assessment of the dynamic characteristics of rocks is a highly intricate process, dependent upon the mechanical properties of the material, microstructural elements, and the nature of the applied loading conditions. Furthermore, the dynamic tensile properties of rocks may exhibit disparities when compared to their dynamic compressive behaviour under varying strain rate conditions^23^. Numerous methodologies are available for elucidating the dynamic behaviour of rocks, and the Split Hopkinson pressure bar (SHPB) stands as a prominent instrument among these diverse approaches. The dynamic strength of rocks is typically quantified using the ‘dynamic increase factor (DIF),’ which represents the relative enhancement of dynamic strength in relation to quasi-static strength. Liu et al.^24^ conducted a comprehensive analysis of dynamic increase factors (DIFs) for a different rock subjected to both compression and tensile loading conditions. They subsequently formulated empirical equations to describe the relationship between dynamic strength and static strength as a function of the applied strain rate. These empirical equations, or dynamic increase factors (DIFs), are specifically applicable to a particular rock type and the testing conditions employed; thus, their applicability is inherently restricted. Kimberley et al.^25^ formulated a universal, rate-dependent scaling law applicable to brittle materials, incorporating microstructural parameters. The Kimberley models have the capability to predict the tensile strength of brittle materials by manipulating certain material characteristics. Nonetheless, the applicability of the Kimberley model to thermally altered rock remains unverified. Moreover, Li et al.^26^ expanded the Kimberley model for predicting tensile strength by employing the Grain-Based Discrete Element Method (GB-DEM) and established an alternative model while retaining the fundamental characteristics intact. The Kimberley model and the Li et al. model will be introduced in the discussion section alongside our experimental findings concerning thermally treated sandstone.

**Figure 1:**
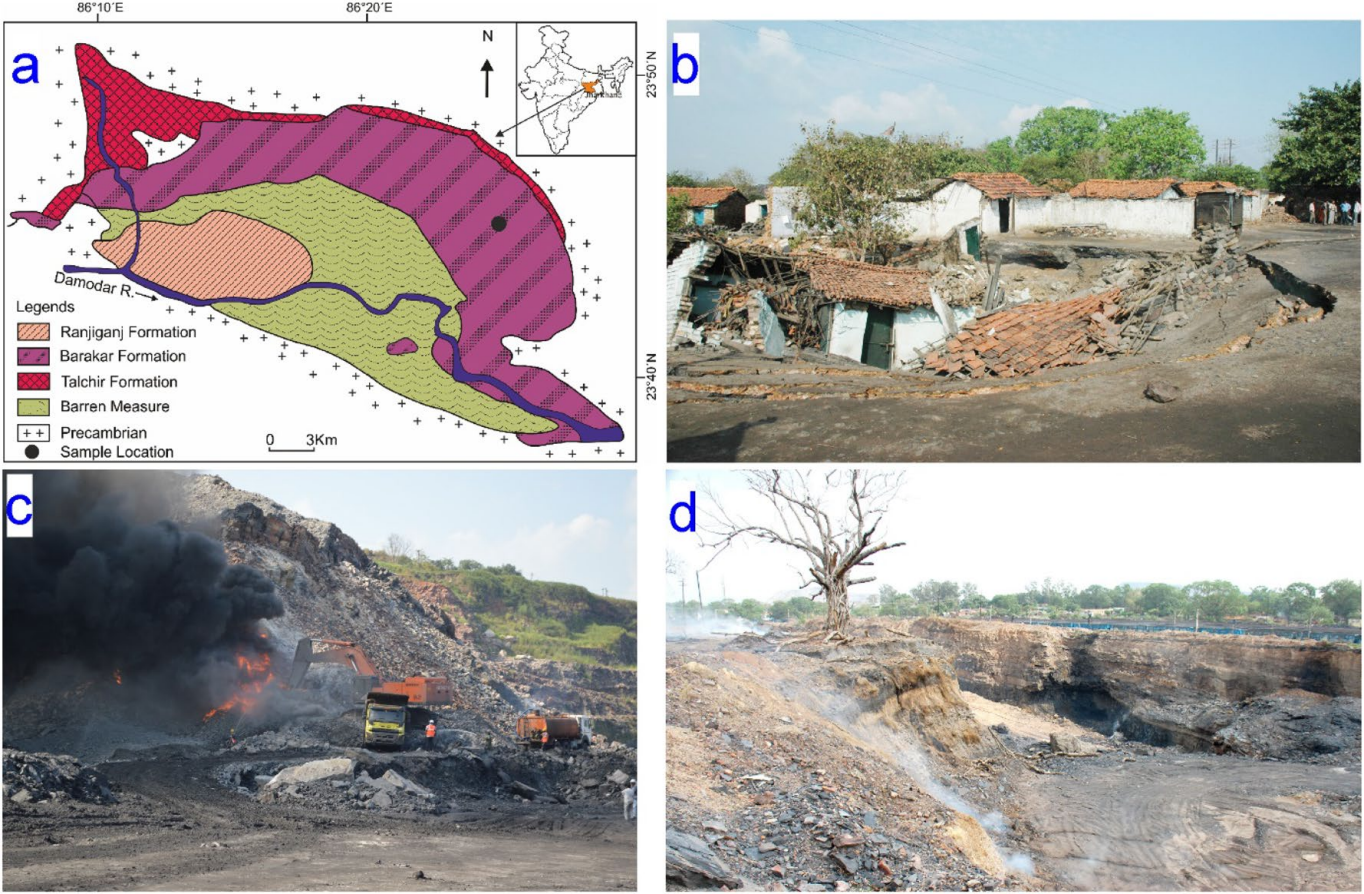
(**a**) Geological map illustrating the spatial arrangement of stratigraphic layers representing colliery rocks within the Jharia Coalfield (adapted from Tripathi et al.^4^). (**b**) Property destruction resulting from land subsidence caused by UCF. (**c**) Difficulties encountered during opencast mining as a result of UCF. (**d**) UCF affected coal-bearing rocks in Jharia region. Courtesy (**b**-**d**) Gautam Dey.

The Jharia Coalfield is known for being one of the world’s largest underground coal mine fire regions. It boasts 23 expansive underground mines and nine sizable open-cast mines. Regrettably, underground coalmine fires (UCF) have had a significant impact on the coalfield (Fig. 1). Its value extends beyond mere coal production, as it constitutes a vital component of the Gondwana coalfields within the Damodar valley of India. These fires, originating in 1916 due to uncontrolled and excessive coal exploration, have wrought havoc. Coal fires can occur within the temperature range of 700–900 °C^27–29^. Blasting, being economical and feasible, is one of the methods of coal mining in this region. It causes dynamic loading on the coalmine fire-affected coal-bearing rocks. Understanding the mechanism of failure of coal-bearing sandstones from an underground coal mine fire area that has suffered severely from land subsidence makes this investigation extremely relevant. As dynamic loading due to blasting is inevitable in coal mine regions, the comparative study of dynamic and quasi-static tensile behaviour would be very useful to understand the role of loading conditions and get insights into the failure responses of coal mine fire-affected coal-bearing sandstones.

Therefore, this study aimed to examine the quasi-static and dynamic tensile strengths of thermally treated Barakar sandstone (BS) samples from the Jharia coal mine fire region. Various temperature conditions, i.e. 25 °C, 200 °C, 400 °C, 600 °C, and 800 °C, were investigated. The quasi-static split tensile strength tests were conducted using a servo-controlled universal testing machine (UTM) to indirectly assess the tensile strength of nine groups of thermally treated specimens. The dynamic tensile strength of the treated sandstone was determined using the Split Hopkinson pressure bar (SHPB) apparatus. Microscopic and mineralogical characteristics were analysed through detailed thin-section studies using a petrological microscope. Additionally, a high-speed camera was utilised to observe the dynamic failure behaviour of sandstone subjected to elevated temperatures. Furthermore, this work confirms the validity of both the Kimberley model and the Li et al. model through the new experimental observations pertaining to thermally treated sandstone reported in this work.

## Methodologies

### Sample preparation and thermal treatment

**Figure 2:**
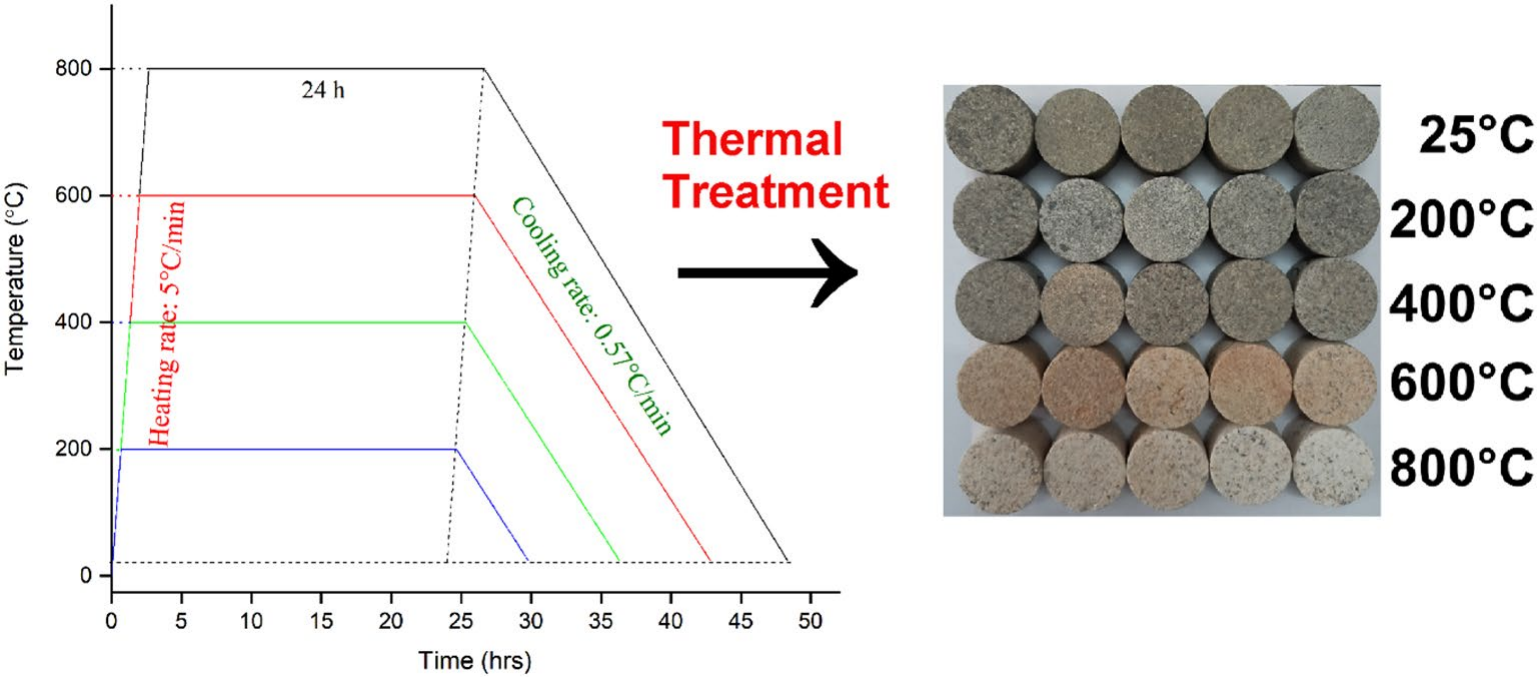
Representative specimens after heat treatment process.

To investigate quasi-static split and dynamic tensile strength, Brazilian disc specimens were prepared according to recommended guidelines^30,31^. The L/D ratio was kept at approximately 1:2 for static and dynamic tests. Generally, smaller specimens easily achieve force equilibrium and higher loading rates for SHPB test^31^. To facilitate the condition of dynamic force equilibrium and the central initiation of cracks, further modifications were made to the cylindrical specimens. Flattering of the ends of the specimens has been recommended to prevent the concentration of compressive stress and failure at the loading end^32–35^. Thus, to make the flat ends parallel to each other, two ends of the specimen that are in contact with the bars were trimmed approximately 0.25 mm to get smooth, flattened ends. It helps to distribute the load from the bar to the specimen over the flattened area. Then the prepared rock specimens were kept in the furnace at a desired temperature for 24 h (heating rate: 5 °C/min) so that the specimens would heat homogeneously. After heating at the desired temperature, specimens were allowed to cool within the furnace (cooling rate: 0.57 °C/min) to minimise the thermal shock (Fig. 2). Specimens subjected to thermal treatment were categorised into five distinct groups, namely 25 °C, 200 °C, 400 °C, 600 °C, and 800 °C. Each temperature group contains three and ten specimens for quasi-static tensile strength and dynamic tensile strength, respectively. These groups were established for the assessment of both quasi-static and dynamic tensile strength.

### Microscopic and mineralogical study

Mineralogical as well as textural changes such as mineral content, mineral shape, and grain-boundary relationship with elevated temperature were investigated through petrographic study. The polished thin sections for each temperature group were prepared and investigated through an optical microscope under plane-polarised and crossed-polarised light. Microscopic characteristics like thermally induced microcracks and grain structures were observed.

### Quasi-static split tensile strength

The quasi-static split tensile strength (${\sigma }_{t}$) tests were performed to indirectly determine the tensile strength of nine groups of thermally treated specimens through UTM, having a capacity of 1000 kN with a controlled loading rate of 200 N/s. The tensile strength of the Brazilian disc can be calculated using Eq. (1):

1 $${\sigma }_{t}= \frac{2P}{\pi Dt}$$

where ${\sigma }_{t}$ is tensile strength of specimens in MPa, *P* is the breaking load in N, *D* is the diameter of specimens in mm, and *t* is the thickness of the Brazilian disc in mm.

### SHPB test apparatus and its working principle

**Figure 3:**
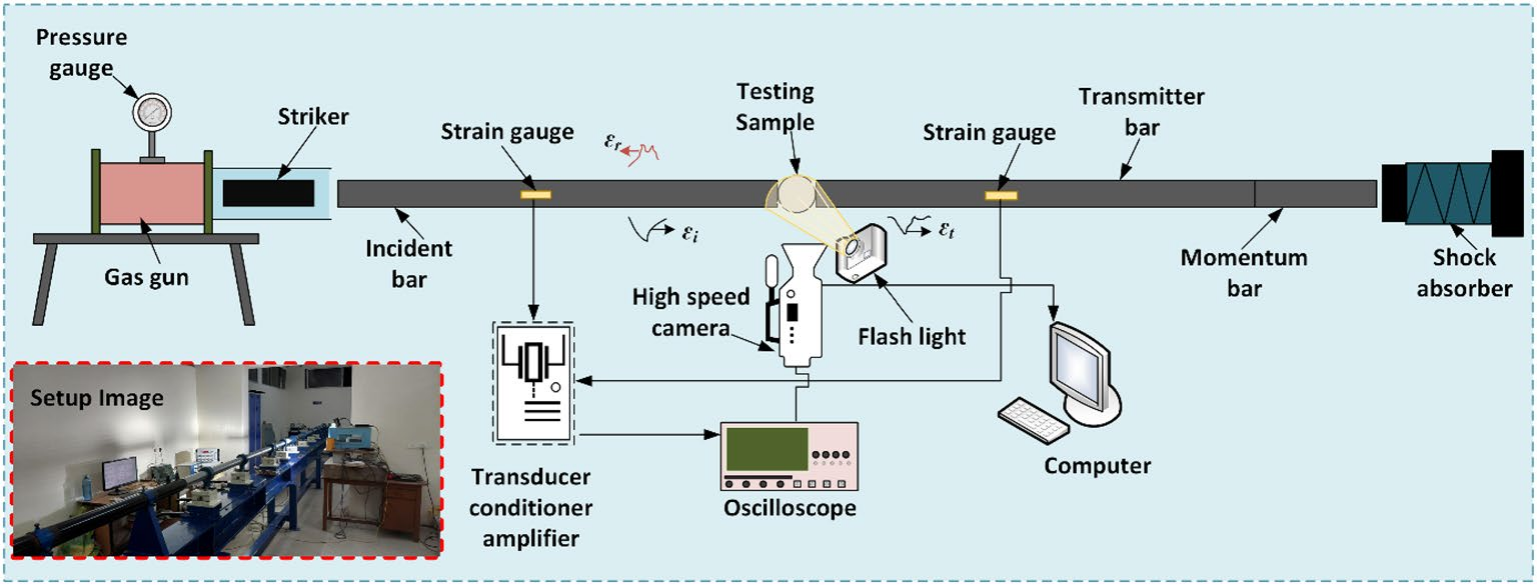
Schematic of Split Hopkinson pressure bar (SHPB) with its component details.

The SHPB, or Kolsky bar setup, principally consists of three main components: (1) Loading components, including the gas gun, barrel, and striker, (2) Bar components, including the incident bar, transmission bar, momentum bar and energy absorber (3) The data acquisition system includes the strain gauges, Wheatstone bridge, amplifier, oscilloscope, and computer system (Fig. 3). The incident and transmission bars have the same geometrical configuration with length (L) 2990 mm and diameter (D) 65 mm, with an L/D ratio of 46:1, while the transmission bar and striker bar have 1500 mm and 200 mm length, respectively, with the same 65 mm diameter. All the bars have a 200 GPa modulus of elasticity (E_b_), 7800 kg/m^3^ density (ρ), and 0.3 poison’s ratio and are made up of steel 4340 to prevent plastic deformation during the experiment.

**Figure 4:**
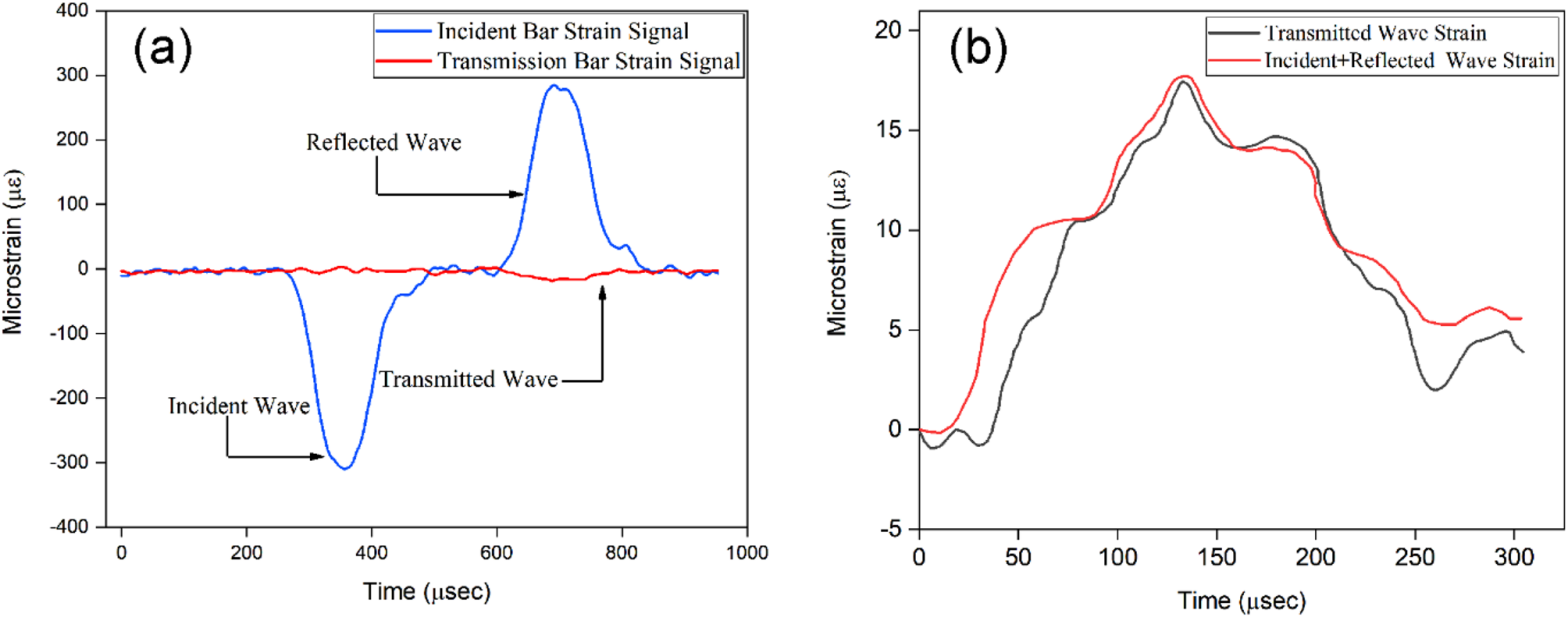
(**a**) Incident bar and transmission bar signal (**b**) Dynamic equilibrium condition.

To determine dynamic tensile strength, the disc specimen is placed in between the incident and transmission bars in such a way that the diameter of the specimen remains parallel to both bars. A longitudinal compressive wave travels in both directions when the free end of the incident bar is impacted by the striker bar. The left propagating wave forms the trailing edge of the incident compressive pulse $\varepsilon_{i}$ (Fig. 3), and is released completely. The length and longitudinal wave velocity affect the duration of loading pulse. Upon arrival at the bare specimen interface, a reflected wave $\varepsilon_{ r}$ is generated as a part of the incident wave, and the remaining part forms the transmitted wave $\varepsilon_ {t}$. The strain wave pulse on both the incident bar and the transmitted bar is recorded by strain gauges (Fig. 4a). For the majority of experiments, it's essential to have knowledge of the separation between the strain gauges and the specimen.

This information is crucial for establishing the initial timing of incident, reflected, and transmitted pulses. Additionally, an optical technique can be employed to measure the velocity of the impacting bar, while the Wheatstone bridge circuit with amplification is commonly utilised for the acquisition of strain signals. The experimentally obtained signal in incident and transmission bars has been illustrated in Fig. 4a. The experimental figure depicted in Fig. 4b demonstrates the attainment of a state of dynamic stress equilibrium within the specimen. The progressive deformation of specimens was continuously recorded by a high-speed camera at 40,000 fps.

Once the dynamic stress equilibrium was achieved, the forces F_1_ and F_2_ acting on the left and right ends of the specimen, respectively, were calculated using Eqs. (2) and (3).

2 $${F}_{1}\left(t\right)= {E}_{b}{A}_{b}\left[{\varepsilon }_{i}\left(t\right)+{\varepsilon }_{r}\left(t\right)\right]$$

3 $${F}_{2}\left(t\right)= {E}_{b}{A}_{b}{\varepsilon }_{t}\left(t\right)$$

where* E*_*b*_ and *A*_*b*_ are Young’s modulus and area of the bar respectively.

The dynamic split tensile strength (${\sigma }_{td}$) of the specimens was calculated using Eq. (4)^36^:

4 $${\sigma }_{td}=\left(\frac{{E}_{b}{D}_{b}^{2}}{2{L}_{0}{D}_{s}}\right){\varepsilon }_{t}^{max}$$

*E*_*b*_ is the Young’s modulus of the pressure bar, *D*_*b*_ and *D*_*s*_ is the diameter of the bar and specimen, respectively, *L*_*0*_ is the thickness of the specimen and *ɛ*_*t*_^*max*^ is the maximum strain measured in the transmitted bar.

Since the strain rate is variable in the loading duration, the average strain rate can be calculated using Eq. (5)^37^:

5 $${\dot{\varepsilon }}\,{ = - }\frac{{\sigma_{td} }}{{E_{s} T}}$$

where $\dot{\varepsilon }$ is the strain rate, *E*_*s*_ is the young’s modulus of elasticity of specimen, and *T* is the time lag between the start of the transmitted wave and maximum occurrence of the transmitted wave.

## Results and discussion

### Effect of elevated temperatures on mineralogical and microscopic characteristics

**Figure 5:**
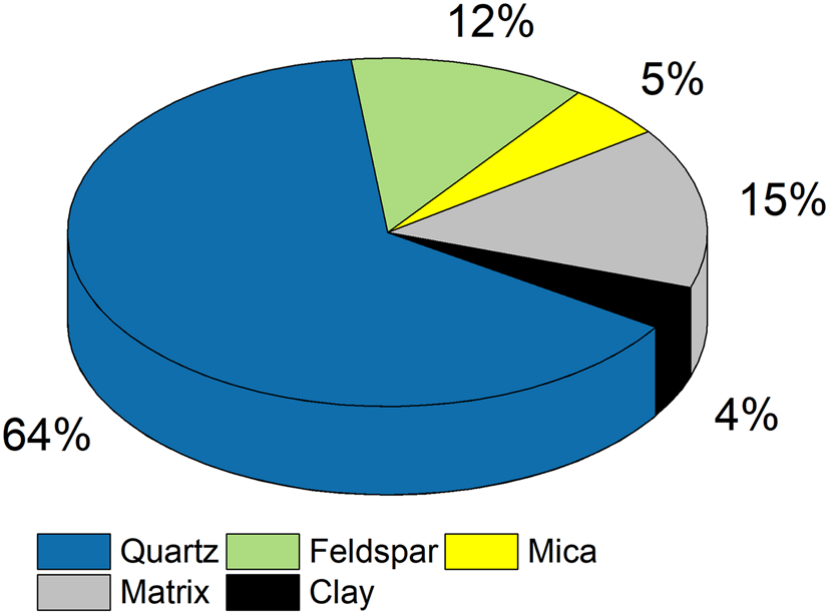
Mineral percentage observed in BS.

**Figure 6:**
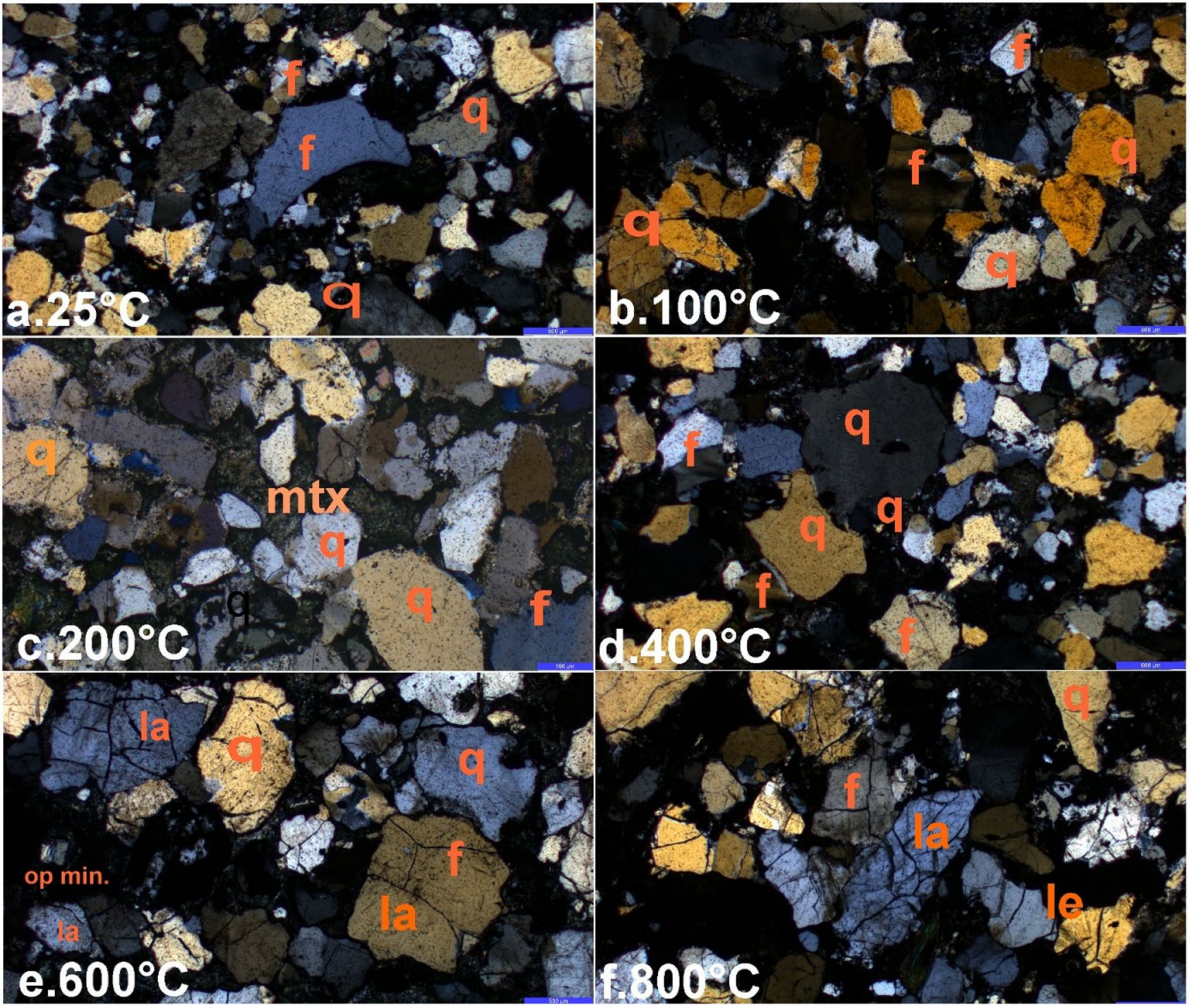
Petrographic images of rock at different temperatures: (**a**–**d**) Thin section study shows rock is majorly composed of quartz, felspar (orthoclase and plagioclase), matrix. (**e**,**f**) At high temperatures (600 °C and 800 °C) more prominent and intense cracks (Ia & Ie) damage the rock. These cracks are either perpendicular or parallel to the main crack with branching results intense grain damage. Q quartz, mtx matrix, f feldspar, op opaque mineral, Ia intragranular cracks, Ie intergranular cracks.

The composition of BS predominantly comprises quartz (64%), feldspar (12%), mica (5%), and clay (4%) with matrix (15%) also referred to as 'wacke' (Fig. 5). The petrographic findings depicted in Fig. 6 showcase various mineralogical and microscopic observations with elevated temperatures. Thin section images confirmed the presence of both fresh and partially weathered feldspar grains, which produce kaolinite and contribute to the sandstone's grey colour. The thin sections of BS exhibited a range of primary and secondary matrix compositions including silty to clayey compositions, along with ferruginous, siliceous, and calcareous types of cement. The mineral grains displayed an angular to subangular shape and varied in size from coarse to medium, indicating limited transportation history prior to deposition (Fig. 6). Additionally, the presence of lithic fragments and opaque minerals was observed (Fig. 6), suggesting that BS is texturally and mineralogically immature.

At 25 °C, the mineral grains demonstrated minimal cracking, except for a few inherent cracks present in the quartz grains (Fig. 6a). The absence of thermally induced microcracks and the preservation of intact grain boundaries indicate that temperature changes up to 400 °C did not cause significant alterations (Fig. 6a–d). However, when the temperature exceeded 400 °C, thermal cracks were observed in microphotographs (Fig. 6e and f). The quartz grains exhibited multiple random thermal cracks, forming a complex network, whereas the feldspar grains opened along their cleavage planes due to thermal expansion (Fig. 6f), which interacted with microcracks and contributed to the formation of a complex fracture network. Most cracks were formed during the heating stage rather than the cooling phase. The structural damage of BS was significantly influenced by the variation in thermal expansion across distinct crystallographic axes within the mineral grains, particularly when the temperature exceeded 400 °C. The Barakar Sandstone mainly comprises quartz (64%) and feldspar (12%). Therefore, quartz, with its four times greater volumetric expansion than feldspar^38^, contributed more to the observed thermal damage. The initiation of cracks preceding grain boundary separation served as an indicator of high thermal stress. The thin section image clearly indicated an increasing fracture density with rising temperature (Fig. 6e and f). Additionally, the α-β quartz transition caused intense cracking at a temperature of 573 °C, as depicted in Fig. 6f. As the temperature increased, both intergranular and intragranular cracks were formed (Fig. 6e and f).

### Effect of elevated temperatures on the mass loss rate

Careful investigation of changes in mass at elevated temperatures provides an idea of internal changes that control the mechanical strength of thermally treated rocks. Evaporation of water from pore spaces, lattice water, burning of organic matter, and some decarbonisation reactions may reduce the mass of rocks. So, the mass loss rate (K_m_) is defined as the ratio of mass loss of a thermally treated specimen (M_T_) to the initial mass (M_I_) of the untreated specimen, expressed in Eq. (9)^39^:

6 $${K}_{m}=\frac{{M}_{I}-{M}_{T}}{{M}_{I}}$$

**Figure 7:**
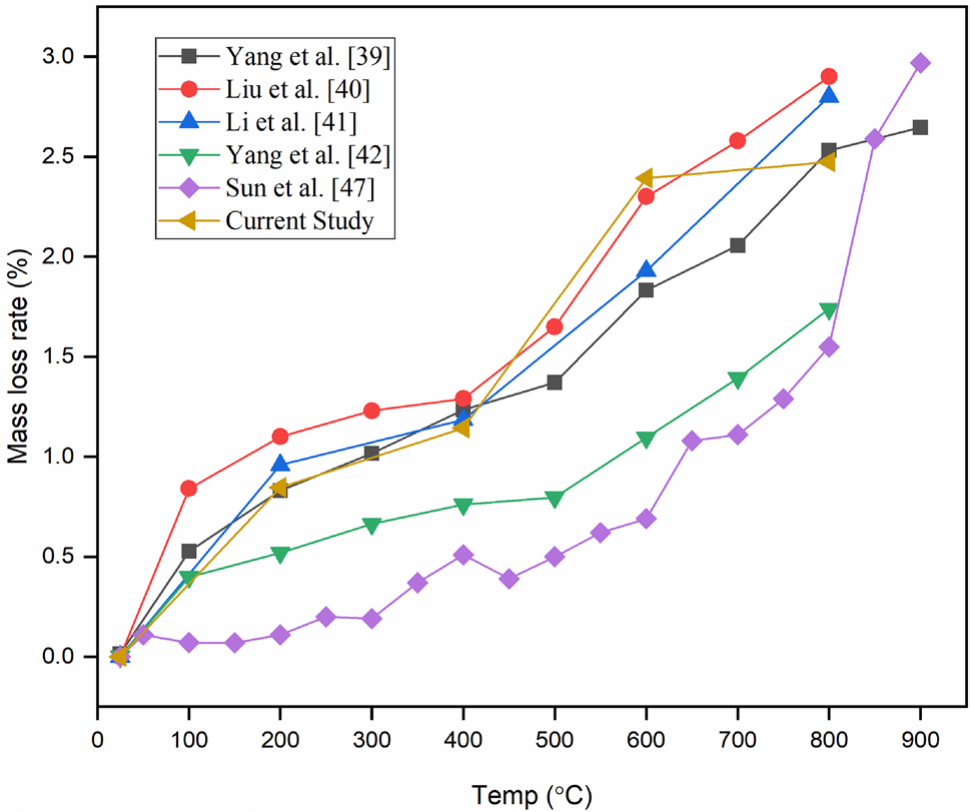
Comparative study of variation in mass loss rate of BS specimen with elevated temperature.

Figure 7 shows variation in the mass loss rate of BS specimens at elevated temperatures and a comparative study with literature data for sandstone. Comparing these new results with previously published works offers varied insights into comprehending the influence of mass loss rate on the tensile strength of thermally treated sandstone under both static and dynamic loading scenarios, which is another novel aspect of the present work. It is observed that the slope of the mass loss curve is steep up to 200 °C due to the escape of absorbed water and partial lattice water at temperatures up to 200 °C. However, within 200–400 °C, the increase in mass loss rate was observed to be gentle, which suggests that the less amount of bonded and crystal waters released in this temperature range (Fig. 7). The observed values have good agreement with the other studies^40–42^. The evaporation of water has a significant contribution to mass loss in rocks in mild temperature regimes.

Absorbed water (free water) completely escapes in the temperature range of 100–110 °C^43,44^. Whereas bonded and crystal waters are progressively released in the temperature range of 100–400 °C^45,46^. The temperature range of 400–600 °C is characterized by a steep increase in mass loss rate (Fig. 7) whose trend is coincided with Liu et al.^40^. As the matrix of BS is mostly composed of clay minerals along with a considerable amount of organic matter^4^. Subsequently, the burning of organic matter and some of the thermal reactions in the rock-forming minerals occurred at this temperature range (for example clay minerals start to decompose progressively after 400 °C) contributing to the high mass loss after 400 °C. Additionally, the percentage of mass loss is also depending on the modal percentage of mineral phases present in the rocks. The very high mass loss rate observed in BS after 400 °C also follows the almost same trend as other studies^40–42,47^ as shown in Fig. 7. After reaching 600 °C, the curve displays an almost constant mass loss rate. This rate is notably lower compared to other temperature ranges because the phase transformation, recrystallization of minerals, and changes associated with the matrix consume less mass of the sandstone^4^. Whereas, in the other study mass loss continuously increases with the elevated temperature (Fig. 7) which might be due to the decomposition of carbonates. Thus, it was inferred that although the studied sandstone rocks belong to the same lithology, the variations in mineralogy and texture yield distinct mass loss curves, which notably influence the rock’s strength.

### Thermal behaviour of quasi-static tensile strength

**Figure 8:**
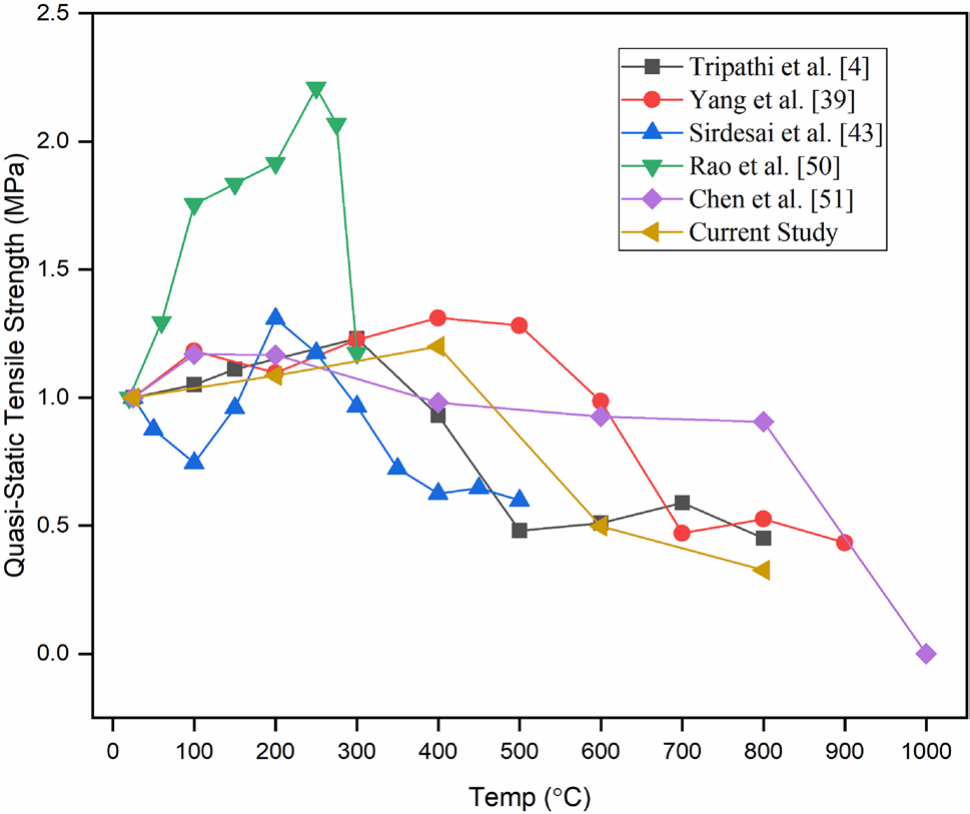
Quasi-static tensile strength variation of thermally treated BS specimen with elevated temperature.

The variation in normalised tensile strength with elevated temperature is shown in Fig. 8. The normalised value of tensile strength has been obtained by dividing the tensile strength at a particular temperature by the tensile strength at room temperature. The normalized tensile strength of sandstone is gently increasing up to 400 °C and thereafter decreasing sharply up to 800 °C (Fig. 8). The continuous increment in tensile strength up to 400 °C is due to the evaporation of different kinds of associated water molecules that provide friction between the mineral grains, resulting in strength enhancement. The thermally induced microcracks were almost absent up to 400 °C, as observed in Fig. 8. The thermal damage does not start to occur until the temperature of the rocks doesn’t reach a particular temperature threshold. Water-related changes mainly affect the mechanical properties of sandstone^48^ as chemical reactions are not operative up to 400 °C^4,49^. The high porosity and microcracks also strengthen the rocks up to 400 °C^50^. Before the generation of new microcracks, the existing microspores and microcracks start to close due to the expansion of grains, lending the rocks a relatively tighter structure^39,43^. This phenomenon provides the observed strengthening of rocks up to 400 °C, as shown in Fig. 8. When all the water-related changes took place and all the micropores and microcracks closed, the newly developed thermally induced microcracks drastically deteriorated the strength of the rock after 400 °C. After 400 °C in this study, the newly developed thermal microcrack and micropores reduce the tensile strength of sandstone from a normalized value of 1.2 to 0.33 up to 800 °C. When all the water-related changes took place and all the micropores and microcracks closed, the newly developed thermally induced microcracks drastically deteriorated the strength of the rock after 400 °C.

After 400 °C in this study, the newly developed thermal microcrack and micropores reduce the tensile strength of sandstone from a normalized value of 1.2 to 0.33 up to 800 °C. This is evident in the photomicrographs of thermally treated BS, where microcracks continuously increase with increasing temperature (Fig. 6e–h). Sirdesai et al.^43^ showed a reduction in the normalised tensile strength of sandstone from 1.17 to 0.60 in the temperature range of 250–500 °C. Rao et al.^50^ found a decrease from 2.21 to 1.17 in the temperature range of 250–300 °C. Whereas, Chen et al.^51^ observed the tensile strength reduction from 1.17 to 0.91 in the temperature range of 200–800 °C. Similarly, Yang et al.^39^ found a reduction of tensile strength from 1.28 to 0.43 within the temperature range of 500–900 °C. In contrast with this, Tripathi et al.^4^ found an almost stabilised trend in the temperature range of 500–800 °C for BS due to partial melting and recrystallization of the matrix. Thus, these findings emphasise the crucial role of temperature in altering sandstone's mechanical properties, highlighting a complex interplay of factors under heat exposure.

### Thermal behaviour of dynamic characteristics

#### Thermal behaviour of dynamic tensile strength

**Figure 9:**
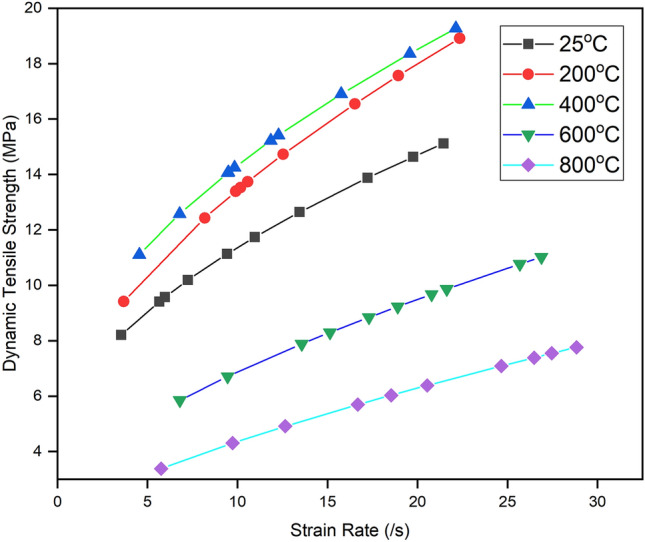
Variation in dynamic tensile strength of thermally treated BS specimen.

The dynamic tensile strength of thermally treated BS was calculated using the methods described in Sect. “SHPB test apparatus and its working principle”. The observed dynamic tensile strength gently increases from 25 to 400 °C. According to the observations, at 400 °C, BS has the highest quasi-static and dynamic tensile strength to withstand deformation. The water has a positive impact on the dynamic tensile strength of BS, similar to the quasi-static tensile strength (Fig. 9). The role of absorbed water, bonded water, crystal water (chemically unbonded), and structural water (chemically bonded) as discussed in Sect. “Thermal behaviour of quasi-static tensile strength” might be the possible cause of strengthening in dynamic tensile strength. The intriguing aspect of this phenomenon lies in the noteworthy correlation between tensile strength and strain rate, particularly within the temperature range up to 400 °C.

The observed strengthening of sandstone with an increasing strain rate can be explained by the thermal activation mechanism^52^. The generation of microcracks occurs as atomic bonds break due to the thermal vibration of atoms. Under low-strain conditions, microcracks have sufficient time to develop along weaker pathways. However, under high-strain conditions, stress waves cannot be transmitted out of the rocks for this short duration. As a result, the entire distribution of flaws may be activated, requiring more atomic bonds to break. This leads to the strengthening of rocks under high rates of loading. The alternative explanation for the observed strength enhancement (Fig. 9) involves the fractured rock moving at accelerated speeds during high strain rates, generating inertial stress along the loading direction. Additionally, lateral inertial stress acts to impede the outward motion of the inner rock mass due to Poisson's ratio, potentially contributing to strength enhancement under high strain conditions.

Furthermore, the strengthening effect is compounded by the evaporation of water and the absence of thermally induced microcracks as the temperature rises to 400 °C. After 400 °C, where the thermally induced cracks are starting to initiate, the dynamic tensile strength begins to sharply decrease with the increasing temperature up to 800 °C. The reduction in dynamic tensile strength is associated with increased porosity and thermally induced cracks (Fig. 6e,f). Hence, the temperature-strengthening effects were seen for dynamic tensile strength in the temperature zone of 25–400 °C. In contrast with this, weakening was observed for dynamic tensile strength in the temperature zone of 400–800 °C. With the increasing temperature, thermally induced microcracks emanated within the grains as well as grain boundaries. The uneven expansion of mineral grains results in increments in thermally induced microcrack density which degrades the dynamic tensile strength. Quartz, a major constituent of sandstone, shows intragranular cracks at high temperatures. Furthermore, it experiences an irreversible α-β phase transition at 573 °C^4^. This phase transition is associated with a volumetric expansion, which could potentially contribute to the formation of intragranular and intergranular microcracks. These microcracks may, in turn, account for the observed reduction in the prominence of strength sensitivity with increasing strain rate within the temperature range spanning from 400 to 800 °C.

#### Dependency of dynamic increase factor on temperature

**Figure 10:**
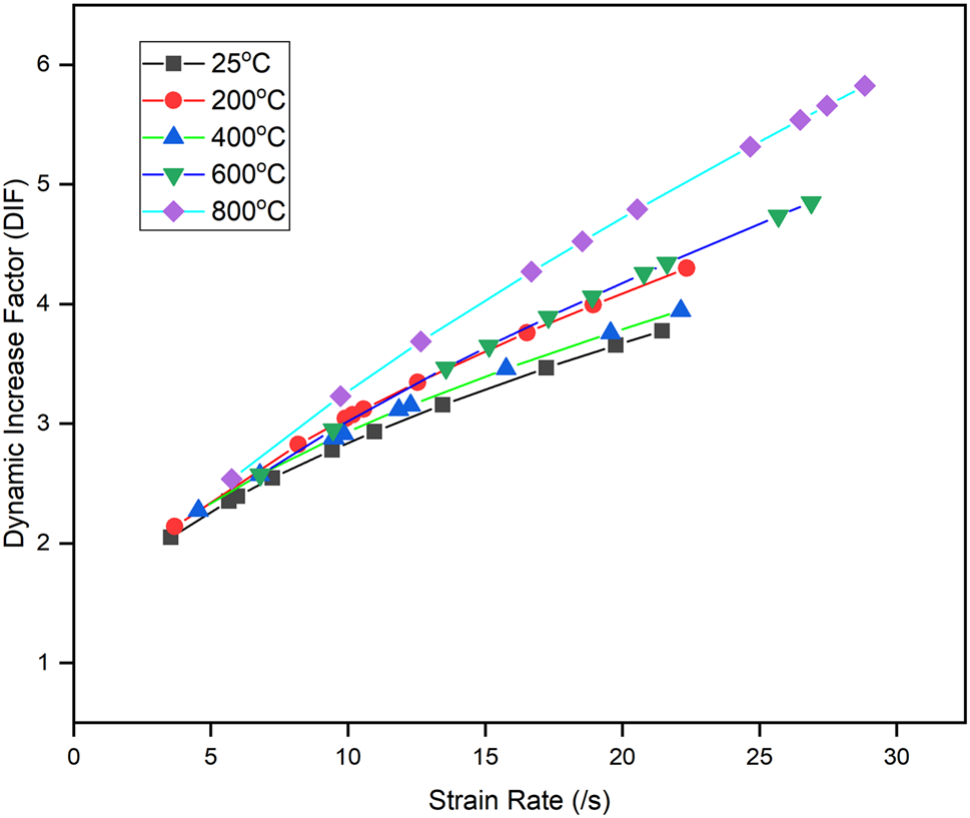
DIF variation of thermally treated BS specimen with elevated temperature.

Dynamic increase factor (DIF) is defined as the ratio of dynamic strength to the quasi-static strength in compression or tension. It is used to understand the rate sensitivity of any brittle materials^24^. The variation of DIF with increasing strain rate and temperature is shown in Fig. 10. The DIF of thermally treated specimens varied between approximately 2 to 6 in the temperature range of 25 to 800 °C. DIF lies between approximately 2 to 4 in the temperature range of 25–400 °C. However, DIF increases from 2 to 6 in the temperature range of 400–800 °C. Since the increment in quasi-static and dynamic tensile strength is almost similar, no change in DIF has been observed up to 400 °C except for 200 °C. The factors operative in mild temperatures are common for an increment in quasi-static and dynamic tensile strength. However, the major chemical and textural changes observed after 400 °C result in the specimen becoming more sensitive to the temperature. The continuous decrement was observed in quasi-static and dynamic tensile strength with increasing temperature. However, quasi-static tensile strength is severely affected as compared to the dynamic tensile strength of sandstone and becomes the reason for increase in the DIF with elevated temperature and stain rate, which increases from 2 to 6 in the temperature range of 400–800 °C.

#### Characteristics strain rate with temperature

Figure 9 illustrates the strain rate dependence of the dynamic tensile strength of thermally treated BS specimens. The dynamic tensile strength of these specimens exhibits a strengthening effect up to a temperature of 400 °C, followed by a subsequent decline. Notably, sandstone, being a sedimentary rock, exhibits a more intricate response with increasing temperature compared to crystalline rocks such as granite, basalt (igneous), quartzite and marble (metamorphic). This complexity arises from the interplay of several factors. Initially, up to a 400 °C, the available pores and pre-existing microcracks in the sandstone tend to close. However, beyond 400 °C, the newly developed microcracks assume a prominent role in causing damage to the sandstone.

The dynamic increase factor (DIF), is known to be influenced by the strain rate or loading rate^23^. Nevertheless, Kimberley et al.^25^ introduced a universal scaling law that incorporates the material's microstructural properties, offering a broader perspective on this model. The model delineates the characteristic stress (*σ*_*0*_) and characteristic strain rate (***ɛ̇˳***) by integrating mechanical parameters such as Young's modulus (*E*), fracture toughness (*K*_*IC*_), p-wave velocity (*v*_*p*_), and microstructural parameters including flaw size (*s̅*) and flaw density (*ƞ*). Equations (7) and (8) delineate the functional expression for characteristic stress and characteristic strain rate as elucidated in the pioneering research by Kimberley et al.^25^.

7 $${\sigma }_{0}=\alpha \frac{{K}_{IC}}{{\overline{s}\eta }^{1/4}}$$

8 $${\dot{\upvarepsilon }}_{0}={\sigma }_{0}\frac{{v}_{p}}{E}{\overline{s}\eta }^{1/4}$$

The characteristic stress is the stress needed to initiate crack formation, effectively spanning the inherent material flaws. The parameter *α* is chosen to make sure that the characteristic stress matches the quasi-static compressive or tensile strength value. The characteristic strain rate (***ɛ̇˳***) is the critical strain rate at which the strength of the rock is twice the quasi-static strength (DIF = 2). Kimberley et al.^25^ proposed a universal theoretical scaling relationship in terms of characteristic stress and characteristic strain rate, is expressed in Eq. (9):

9 $$\frac{{\sigma }_{t}}{{\sigma }_{0}}=1+ {\left(\frac{\dot{\varepsilon }}{{\dot{\varepsilon }}_{0}}\right)}^{2/3}$$

Numerous studies involving brittle materials such as ceramics and geological substances were conducted under both compressive and tensile conditions^23,25,53^. It has been observed that Kimberley et al.’s theoretical model demonstrates a suitable fit at ambient temperatures. However, it is worth noting that Li et al.^54^ raised concerns regarding the applicability of Kimberley’s model, particularly in the context of tensile loading. In response to these concerns, Li et al.^54^ introduced an alternative model that bears similarities to Kimberley's model, developed through numerical simulation (DEM). Additionally, they proposed a more foundational formulation (Eq. 10) for the DIF.

10 $$\frac{{\sigma }_{t}}{{\sigma }_{0}}{=1+\left(\frac{\dot{\varepsilon }}{{\dot{\varepsilon }}_{0}}\right)}^{\beta }$$

In the equation provided above, the variable *'β'* represents a freely adjustable parameter with a range spanning from 0 to 1. In their comprehensive analysis of experimental data, Li et al.^26^ determined that the values of *'β'* exhibit variability within the range of 0.35 to 0.63.

**Figure 11:**
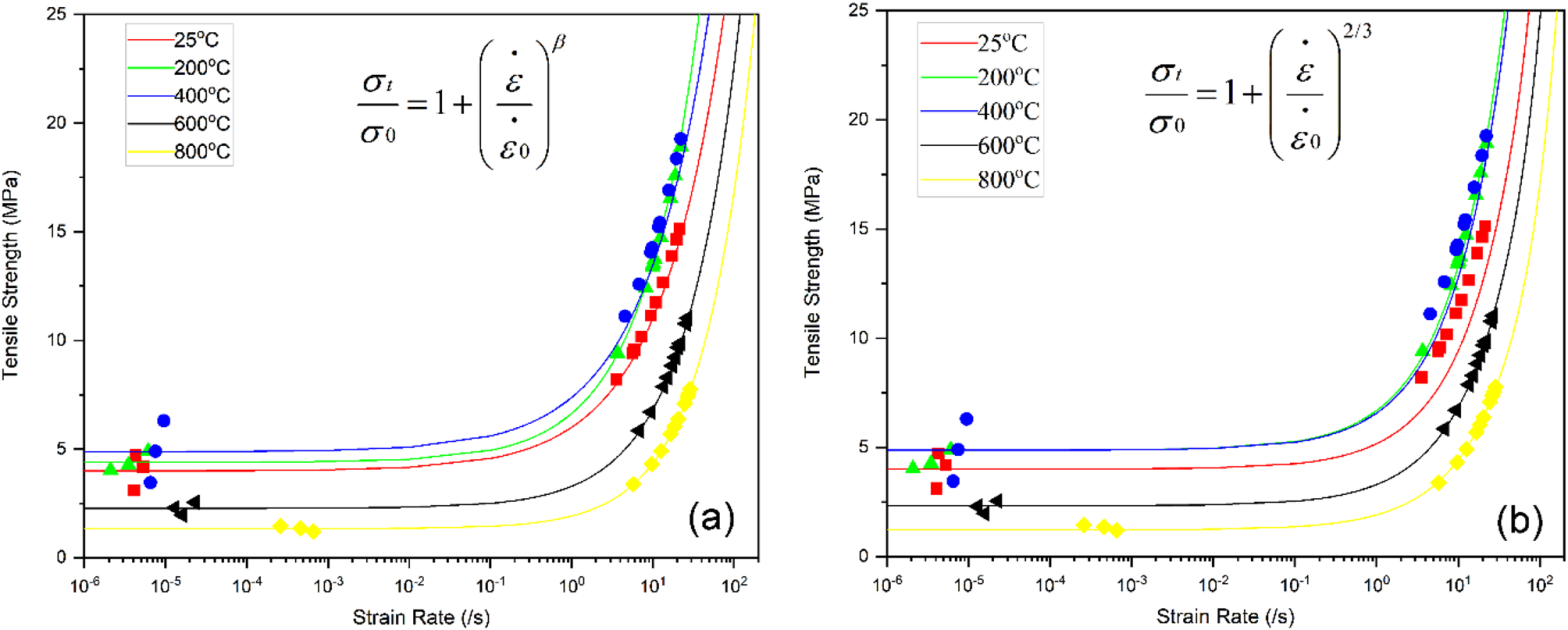
Tensile strength from quasi-static and dynamic testing at various strain rates. Each suit follows the universal scaling relationship of (**a**) Li et al.^54^ and (**b**). Kimberley et al.^25^.

In this current investigation, the free parameter (*β*) and the characteristic stress and characteristic strain of the BS specimen at various temperatures were determined through nonlinear least-squares fitting based on the Li et al.^54^ model (Fig. 11a). The free parameter ‘*β*’ exhibited a range of variation between 0.54 and 0.71. Simultaneously, the characteristic strain rate (*ɛ̇˳*) for the examined BS specimen exhibited values of 3.56 ± 2.16/s, 3.0 ± 0.95/s, 3.48 ± 2.97/s, 3.46 ± 1.15/s, and 3.16 ± 0.69/s at temperatures of 25 °C, 200 °C, 400 °C, 600 °C, and 800 °C, respectively. The characteristic stress (*σ*_*0*_) values were 3.99 ± 0.81 MPa, 4.39 ± 0.44 MPa, 4.88 ± 1.41 MPa, 2.27 ± 0.29 MPa, and 1.33 ± 0.12 MPa for temperatures of 25 °C, 200 °C, 400 °C, 600 °C, and 800 °C, respectively.

Kimberley’s theoretical model is presented in normalised form as the ratio of dynamic strength to quasi-static strength. Initially proposed by Kimberley, the value of *'β'* was set at 2/3 (Fig. 11b). Subsequently, we conducted a repeat curve fitting procedure, resulting in the determination of the definitive characteristic strain rates (***ɛ̇˳***), which were determined as 6.37 ± 1.21/s, 4.43 ± 0.51/s, 6.0 ± 1.93/s, 3.71 ± 0.59/s, and 2.35 ± 0.26/s at temperatures of 25 °C, 200 °C, 400 °C, 600 °C, and 800 °C, respectively. The characteristic stress (*σ*_*0*_) values are 4.73 ± 0.38 MPa, 4.88 ± 0.25 MPa, 5.79 ± 0.80 MPa, 2.33 ± 0.18 MPa, and 1.22 ± 0.73 MPa for temperatures of 25 °C, 200 °C, 400 °C, 600 °C, and 800 °C, respectively.

Based on the Kimberley models, it was concluded that the characteristic strain rate (***ɛ̇˳)*** exhibits variation with increasing temperature. Upon rearranging Eqs. (7) and (8), the characteristic strain rate (***ɛ̇˳***) can be expressed as follows:

11 $${\dot{\varepsilon }}_{0}={\sigma }_{0}\frac{{v}_{p}}{E}{\eta }^{1/2}$$

The above equation clearly illustrates that the characteristic strain rate is contingent upon various factors, including the characteristic stress, p-wave velocity, Young’s modulus, and the flaw density of rocks. Extensive research has shown that the flaw density of rocks undergoes alterations as temperature increases. Furthermore, empirical testing has unveiled significant variations in the characteristic strain rate.

Below 400 °C, there are no significant variations observed in the characteristic strain rate. Furthermore, the absence of thermally induced microcracks has a minimal impact on the characteristic strain rate and other related parameters. However, after 400 °C, there is a gradual and continuous decline in the characteristic strain rate. This reduction appears to be correlated with alterations in characteristic stress, p-wave velocity, Young’s modulus, and flaw density, all of which interact with temperature. Identifying the specific parameter or combination of parameters responsible for this decline in the characteristic strain rate poses a challenge. Nevertheless, this study provides conclusive evidence of the substantial impact of rising temperatures on the characteristic strain rate. The determination of flaw density, P-wave velocity, and elastic parameters in thermally damaged rocks would provide greater insight into the changes in the characteristic strain of thermally treated sandstone, which lies within the scope of our future research.

### Effect of elevated temperatures on the dynamic tensile failure behaviours

**Figure 12:**
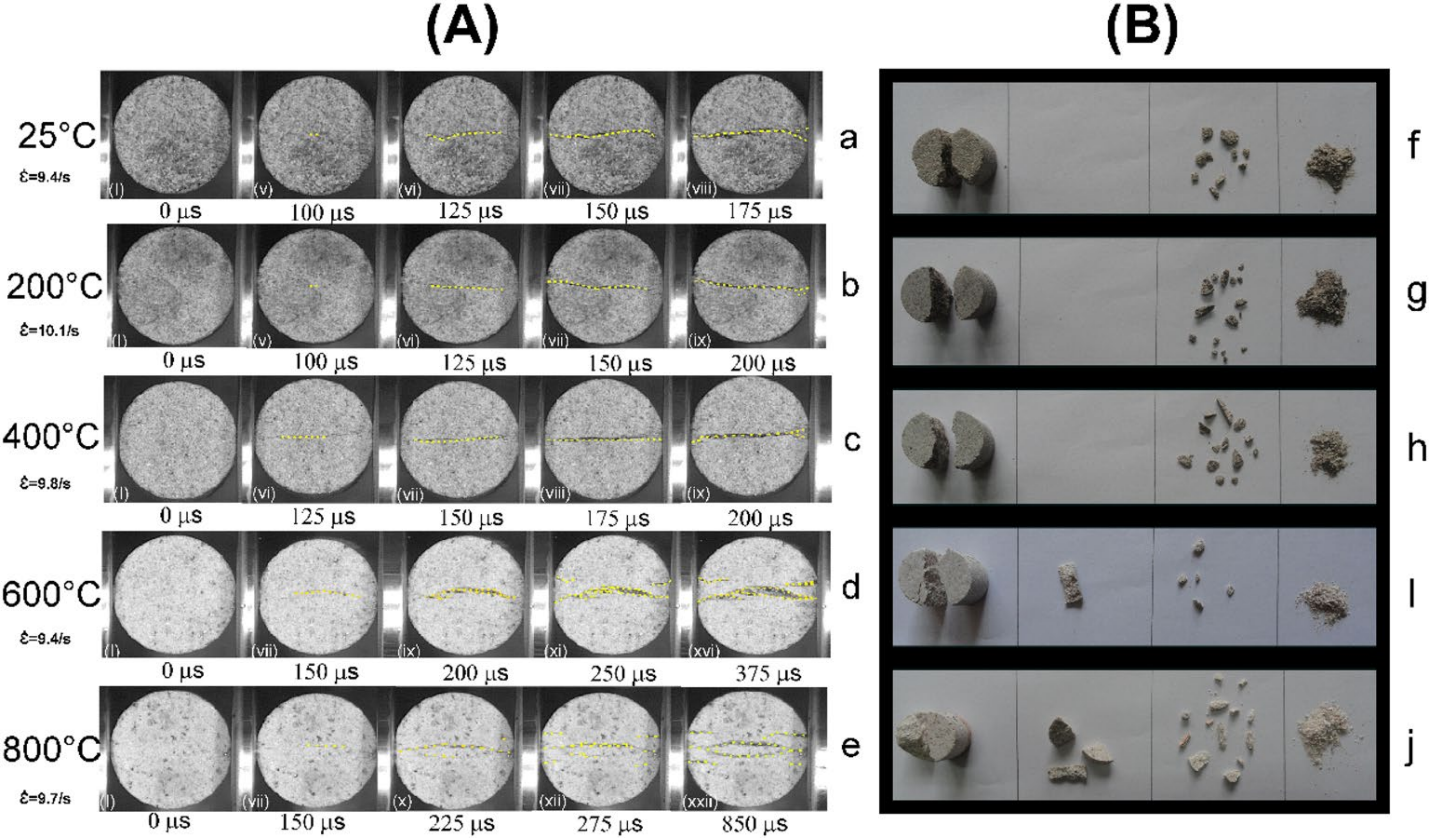
(**A**) Dynamic tensile failure behaviours of thermally treated sandstone. The yellow dotted curves (in the figure above) trace the crack evolution as response to elapsed time under uniform experimental temperature conditions. (**B**) The morphology of fragments after dynamic tensile failure at the respective temperature.

The dynamic failure characteristics of the thermally treated BS were continuously recorded by a high-speed camera at 40,000 fps. Time-series photographs of crack initiation and crack growth within the thermally treated BS were analysed to understand the failure mechanism (Fig. 12A). The validity of the test was confirmed by the observed crack initiation and propagation in the loading direction, which occurred near the centre of the specimen and bilaterally propagated towards the loading ends at each temperature^55^. The observations on the dynamic failure mode suggest that the temperature influences the crack growth at a similar striker velocity.

The first frame (I) represents the initial stage of sandstone specimens, after which the incident wave first appeared at the contact interface between the incident bar and the specimens. The first crack was generated at a time of 100 µs to 150 µs (V-VI frame). A tensile crack was generated and propagated towards the loading direction (Fig. 12Aa–e). Irregular tensile cracks were formed due to grain boundaries, micropores, and pre-existing microcracks^56^. When the temperature is below 400 °C, no loss of cohesion results in the specimens splitting into approximately two halves with fewer fragments. Figure 12A depicts that tensile failure is a prominent failure mode in the loading direction. In mild temperature zone, only water evaporates. However, no thermally-induced microcracks were seen in the thin section. Thus, water doesn’t affect the failure mode up to a temperature of 400 °C. However, after 400 °C, the generated tensile cracks showed a relatively complex pattern due to the coupling effect of thermally induced microcracks and the impact load. The first crack appeared at a time of 150 µs (VII frame) for the temperature range of 400–800°C. This implies that the time of appearing visible first crack is increased due to developed micropores and thermally induced microcracks under high temperature (Fig. 12Ad&e), which enhances the plasticity of BS^56^.

Figure 12Ad&e also suggests that the tensile cracks began at the centre and subsequently propagated in the loading direction, producing a high degree of breakage where the dominant failure mode is still tensile. Careful petrographic investigation reveals that after 400 °C, thermally induced cracks start to initiate and become more prominent with increasing temperature. As a result of dominant thermal cracking after 400 °C, the shear cracks with multiple branching are observed to start after 400 °C and become significant with increasing temperature up to 800 °C (Fig. 12Ad&e). Xi et al.^56^ observed the formation of shear cracks at high temperatures due to the reduction of cohesion between mineral particles, which are prone to failure and relative slip. Although these shear cracks were not in symmetry at low temperatures, at high temperatures, they became symmetrical with the main tensile crack. Under high temperatures, it produces a greater number of fragments, as observed in Fig. 12Bi and j.

## Conclusions

The present study examined the effect of mild to high temperatures on the dynamic and quasi-static tensile behaviours of Barakar sandstone. The new results reported in this work offer additional insights towards understanding the influence of mass loss rate on the tensile strength of thermally treated sandstone under both quasi-static and dynamic loading scenarios. The specimens underwent thermal treatment within a predefined temperature range of 25 to 800 °C for 24 h and were cooled within the furnace to avoid thermal shock. The study yielded the following findings:

- Up to 400 °C, both dynamic and quasi-static strengths showed an increase due to the evaporation of water, which increased the friction between the grains. However, after 400 °C, both strengths decreased drastically with increasing temperature up to 800 °C. Furthermore, the dynamic tensile strength demonstrates sensitivity to strain rate up to 400 °C, albeit with a slight reduction in strain rate sensitivity beyond this temperature point.
- The study suggests that the dynamic increase factor did not change up to 400 °C, indicating that water did not affect these parameters in this temperature zone. However, these factors increased drastically after 400 °C due to the thermal softening of the Barakar sandstone.
- The findings indicate that the dynamic tensile strength of thermally treated BS specimens adheres to the universal scaling law proposed by Kimberley et al.^25^ and Li et al.^54^. Moreover, the study identified characteristic strain rates (${\dot{\varepsilon }}_{0}$) at various temperatures: 6.37 ± 1.21/s at 25 °C, 4.43 ± 0.51/s at 200 °C, 6.0 ± 1.93/s at 400 °C, 3.71 ± 0.59/s at 600 °C, and 2.35 ± 0.26/s at 800 °C. The research provides clear evidence that the characteristic strain rate remains relatively stable up to 400 °C but starts decreasing beyond this temperature threshold. This experimental study represents the first attempt to validate the Kimberley model specifically for thermally treated rocks.
- Temperature affects the tensile failure modes in the observed temperature range. Up to 400 °C, the specimens split into two halves, with fewer fragments having almost single tensile cracks. No thermal microcracks were observed in petrographic analyses, indicating that the water did not affect the failure mode up to 400 °C. However, after 400 °C, the failure mode was relatively complex due to the coupling effect of thermally induced microcracks and impact load. Tensile cracks began at the centre and subsequently, several shear cracks emerged and propagated in the loading direction, producing a comparatively high degree of fragmentation. This study also demonstrates that shear failure does not only depend on the loading rate and that temperature can also change its failure mode under dynamic loading.

## Data Availability

All experimental data generated during the current study are available from the corresponding author on reasonable request.
